# The Editor's Letter-Box

**Published:** 1914-04-11

**Authors:** 


### THE EDITOR'S LETTER-BOX.

### Salaries of Poor-Law Medical Officers.

*To the Editor of* The Hospital.

Sir,—The question you raise is one touching a grave injustice to our younger colleagues. Unfortunately, it is one which those who could do anything are not anxious to attack. There is nothing to be made out of it from an electioneering point of view, and the path of least resistance is easy!

Yet there are few who do not admit, when the matter is forced on their notice, that there is something very wrong with a system which allows the victims to say with truth, even though it is only a bitter jest, "Oh! yes, the Council compels us to retire on marriage, but we can get engaged any number of times." As a matter of fact, the Council carefully selects women reputed to be above the average, the very ones who ought to be wives, pays them good salaries, and tells them that if they venture to marry they will be dismissed as though they had done something disgraceful.

What good purpose can be served by such a policy? Work is never well done if performed under a feeling of endured injustice, such as all these women must experience.

They probably do not wish to marry in many cases. Dislike of any idea of matrimony seems a sign of this generation of women, but the knowledge that they must not think of it if they desire to keep on with their life-work is bound to rankle in their minds.

Moreover, we are rearing up a race of celebate inspectors—excellent scientists doubtless, but in many cases lacking that touch of nature which makes the whole world kin. To come to humble details, surely a married medical woman with a household of her own is more fitted to deal sympathetically with mothers bringing their children up for inspection than the most brilliant spinster, who probably lives in rooms or a club and has no family life at all.

The present attitude of the Council to the married medical women in its service is positively Gilbertian. They may be on the lecturing staff and without impropriety instruct mothers as to how to keep their offspring in health. They may on no account inspect such children with a view to finding out if such teaching is bearing fruit!

The root of the trouble seems to be an ingrained belief that a married woman's life must be one of continuous ill-health, and it is a point of view that one has to combat daily. Should we have anything like the amount of infantile mortality amongst the poorer classes were it not an accepted belief that it is a normal thing for a mother to be always ailing? The Council admits the excellent work done by married women teachers.

If matrimony does not preclude a woman from teaching, why should it do so in the case of inspecting? The work is much less strenuous. Each case should be judged on its own merits. If a married woman be unable to do her work efficiently, get rid of her by all means. If the contrary, she will be a more valuable public servant than before, for she is all that she was, *plus* that knowledge of life and breadth of character which the married state—happy or the reverse—is bound to bring.

It is positively pathetic to an older medical woman, and one who has long been happily married, to see bright young girls, her juniors at hospital, absolutely condemned to celibacy, unless they submit to being coerced into giving up their work entirely. An ex-full-time inspector is, as a rule, unfitted to start in general practice on her own account, as she has had little or no experience of that class of work—as assistant, etc.—so resignation from the Council service means cessation of all medical work—an enormous sacrifice, and one most undesirable for the country, her husband and herself, if made unwillingly.

What is the L.C.C. leading these girls to? They are happy in their work now. Some, perhaps, are engaged and think to marry "some day." Meantime their youth is passing. Ten years hence will they consider that £400 a year in the Council service compensates them for delayed wifehood, and probably motherhood foregone for ever?—Yours, etc., A Woman General Practioner.

April 5, 1914.

[Our readers will notice that both correspondents insist that each case should be decided on its merits, leaving the employer as free as ever to dismiss an unsatisfactory officer. If any of our readers can explain what objection there is to this argument, we shall be glad to publish their views.—Ed. The Hospital.]

